# Aortic root abscess in a high-risk case requiring modified hemi-UFO procedure with custom-made pericardial conduit – a case report

**DOI:** 10.1186/s13019-026-04318-z

**Published:** 2026-06-05

**Authors:** Vanessa I. T. Zwaans, Jasper Iske, Leonard Pitts, Christoph T. Starck, Herko Grubitzsch, Jörg Kempfert, Volkmar Falk, Leonhard Wert

**Affiliations:** 1 Department of Cardiothoracic and Vascular Surgery, Deutsches Herzzentrum der Charité (DHZC), Augustenburger Platz 1, Berlin, 13353 Germany; 2 DZHK (German Center for Cardiovascular Research), Partner site Berlin, Berlin, Germany; 3 Department of Cardiothoracic Surgery, Charité − Universitätsmedizin Berlin, Corporate member of Freie Universität Berlin and Humboldt-Universität zu Berlin, Charitéplatz 1, Berlin, 10117 Germany

## Abstract

**Background:**

The UFO procedure is an established surgical technique to treat extensive endocarditis of the aortic or mitral valve with involvement of the intervalvular fibrous body (IVFB). This technique can be used for radical resection of the whole infected tissue. Independently of the size of the infected region it is unavoidable to replace both valves. In this high-risk re-do case we performed a modified so-called hemi-UFO procedure with preservation of the mitral valve.

**Case summary:**

We present a 71-year-old male patient initially diagnosed with severe stenosis of the aortic valve. An aortic valve replacement with a 23-mm prosthesis was performed via partial upper mini-sternotomy. Eight weeks after surgery an echocardiogram revealed a large vegetation and severe regurgitation of the aortic valve prosthesis. The risk of death following reintervention heart surgery (EuroSCORE II) was calculated as 50.64%. We performed a re-do with full sternotomy. Intraoperatively it was observed that the aortic prosthesis was partially torn out. The aortic annulus exhibited circular infection with an abscess connecting to the left atrium. The tissue of the left atrial roof was partially destroyed, similar to a phlegmonous infection. We opened the left atrial roof and radically resected the infected tissue up to the IVFB. We prepared a custom-made conduit prosthesis of bovine pericardium with a 25-mm valve prosthesis. We replaced two thirds of the ascending aorta with re-implantation of the coronary arteries using the Bentall-de Bono technique. We were able to stabilise and implant the new aortic valve prosthesis with sutures through the opened left atrial roof. The stitches began close to the anterior mitral leaflet region and ended in the direction of the left ventricle outflow tract. All sutures were pericardium-pledgeted and were passed through a bovine pericardial patch. This patch formed a new mitral annulus and was used for the closure of the left atrial roof. We had to reconstruct the IVFB, the roof of the left atrium and the mitral annulus in the anterior (A1), middle (A2) and posterior (A3) segments. The patient was transferred to the intensive care unit with no inotropes and in sinus rhythm. The 3-year follow-up was uneventful.

**Conclusion:**

We showed a successful surgical treatment of aortic prosthesis endocarditis with involvement of the IVFB. We were able to perform a radical resection of the infected tissue, reconstruct and replaced all sacrificed tissue with biological tissue and preserved the native mitral valve in a modified hemi-UFO procedure.

**Supplementary Information:**

The online version contains supplementary material available at 10.1186/s13019-026-04318-z.

## Introduction

The UFO procedure, also known as “Commando operation” or “Combat procedure”, was first mentioned by Manouguian in 1976. It was initially designed to enable enlargement of very small aortic annuli [1]. The abbreviation UFO is not a clinical term. It was mentioned by a surgeon at the Toronto General Hospital who observed this complex technique and compared it with an “unidentified flying object".

This surgical technique has proven successful in patients with double-valve endocarditis with or without an abscess in the intervalvular fibrous body (IVFB) [2, 3].

In patients with aortic valve endocarditis and an abscess in the IVFB, a UFO procedure can be useful. The procedure requires radically resecting all infected tissue and setting the new sutures in healthy tissue for adequate stability. This surgical strategy requires replacement of the aortic and mitral valves in a transaortic approach through the aortic annulus and left atrial roof. The reconstruction of the IVFB, the aortic and mitral annuli and the left atrial roof is performed in a double-patch technique with bovine pericardium [2].

## Case presentation

### Patient details

On October 1st, 2022, a 71-year-old male patient (175 cm, 90 kg, BMI 29.4 kg/m^2^) presented with severe aortic stenosis (aortic valve area 0.7 cm^2^, mean pressure gradient 45 mmHg, peak jet velocity 4.1 m/s). Aortic valve replacement (23 mm) via partial upper mini-sternotomy was subsequently performed. On the 11th postoperative day, the patient needed a cardiac catheterisation with implantation of a drug-eluting stent in the first diagonal branch. In addition, the postoperative course was delayed due to an infection with COVID-19. On the 12th postoperative day the patient was transferred to a local hospital. Five weeks after surgery, the patient presented with shivering, fever and elevated C-reactive protein and leucocytes. Blood cultures tested positive for *Staphylococcus epidermidis*. Antibiotic therapy was adjusted to gentamicin, rifampicin and vancomycin. A transthoracic echocardiography was subsequently performed and showed endocarditis of the aortic valve prosthesis with large floating vegetations causing severe aortic regurgitation and stenosis (Video 1). A computed tomography (CT) confirmed circular endocarditis of the aortic prosthesis with signs of cardiac decompensation like pleural effusion on both sides (Video 2).

### Surgical procedure

After induction of anaesthesia, re-sternotomy with enlargement of the partial upper mini-sternotomy was performed.

**Fig. 1:**
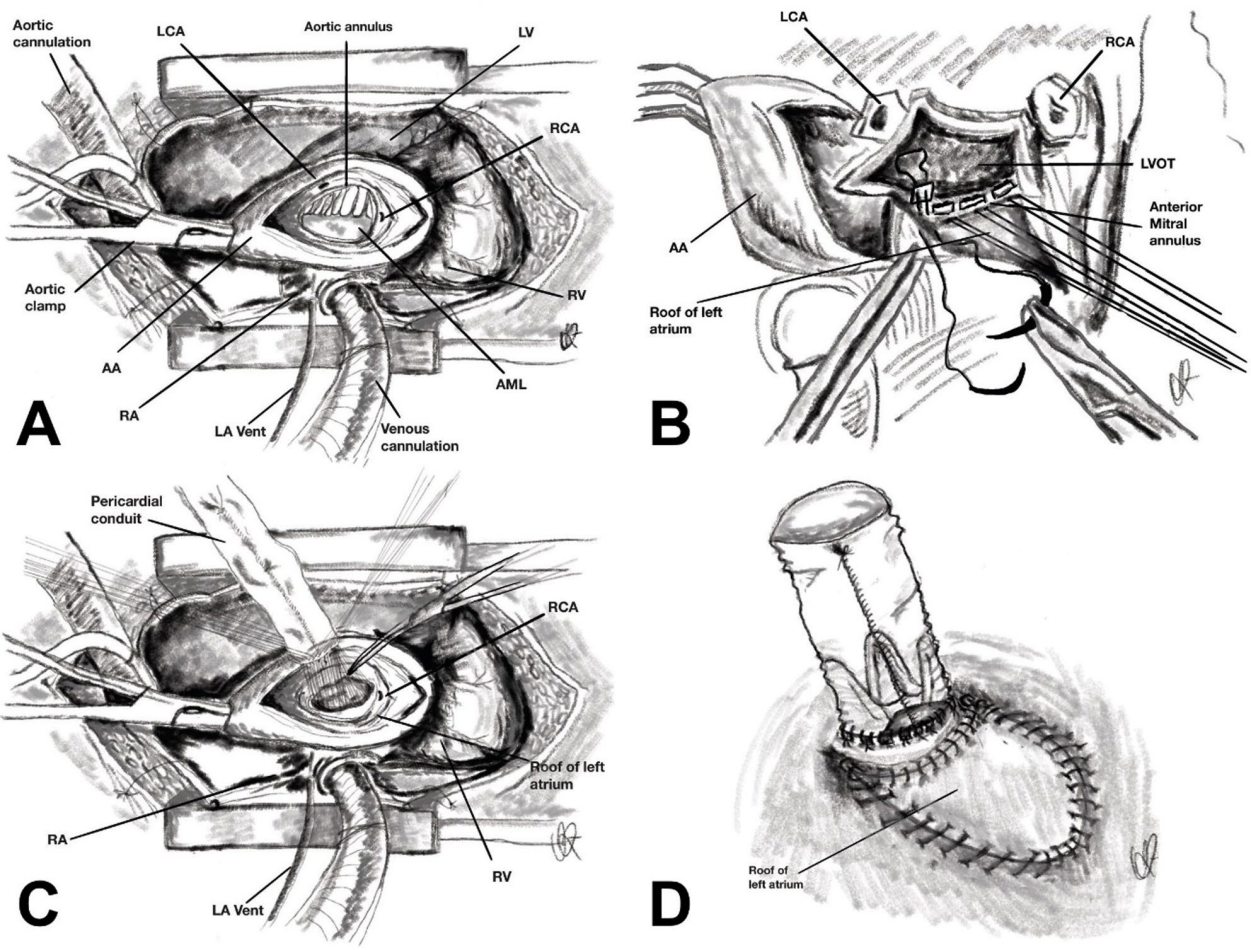
Schematic figure of intraoperative steps. View into the left ventricular outflow tract with parts of the anterior mitral valve after removal of the aortic prosthesis and resection of the aortic root (**A**). Placement of pledged sutures in the anterior mitral annulus after resection of the intervalvular fibrous body (**B**). Parachuting of the composite graft onto the aortic annulus (**C**). Visualization of the closed left atrial roof with implanted composite graft (**D**). AA Ascending aorta, AML Anterior mitral leaflet, LA Left atrium, LCA Left coronary artery, LV Left ventricle, LVOT Left ventricular outflow tract, RA Right atrium, RCA Right coronary artery, RV Right ventricle

**Fig. 2:**
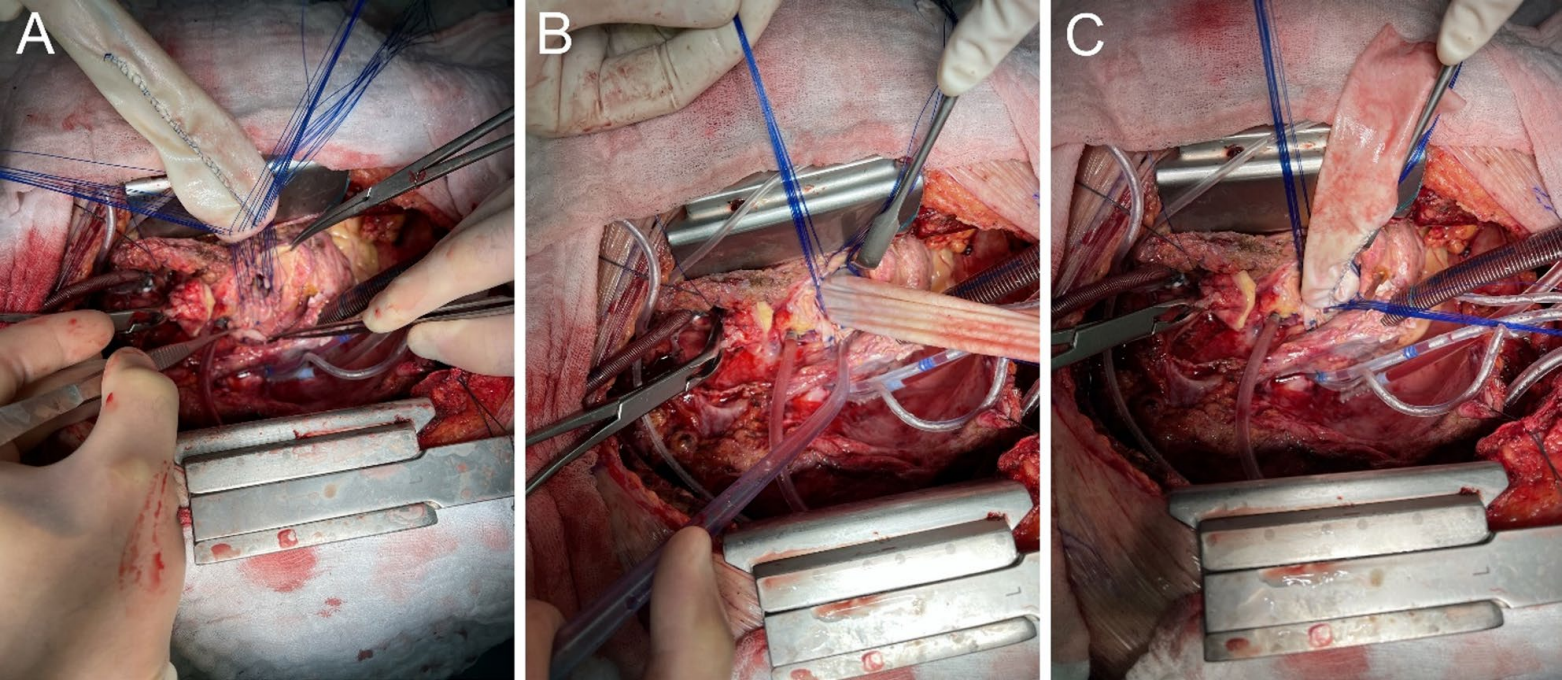
Intraoperative view. Implantation of the custom-made biological composite graft. Surgical step before the custom-made biological composite graft parachutes onto the aortic annulus. Visualization of the closed left atrial roof and direct view into the left ventricular outflow tract. (**A**) Surgical step after parachuting the composite graft onto the aortic annulus. Next step is the knotting of the placed sutures on the prosthesis ring (**B** and **C**)

We placed the arterial line in the aortic arch and a two-stage venous cannula in the right atrium. Cardiopulmonary bypass was established. The operation was performed in normothermia. The ascending aorta was cross-clamped and cardioplegia was administered directly into the coronary ostia. The aortic root was mobilised and circumferentially resected. The coronary buttons were prepared and cut out. We confirmed the circular abscess of the aortic annulus which caused a subtotal avulsion of the aortic valve prothesis. We resected the infected prosthesis and carried out extensive debridement of the damaged tissue. The damaged tissue extended from the ascending aorta to the left ventricular outflow tract (LVOT) including the aortic annulus and the IVFB. By resecting parts of the left atrial roof, the debridement of the damaged tissue between the non-coronary and the left coronary sinuses of Valsalva exposed the anterior mitral leaflet (AML) (Fig. 1A). We confirmed the preoperative echocardiography with no infection of the mitral leaflets and the subvalvular apparatus. The AML was vertically exposed to the surgeon. The mitral annulus exhibited annular tissue deficiency. However, the leaflets of the mitral valve were not affected by the tissue-destroying endocarditis process. We deliberately abstained from resecting mitral annular tissue to avoid atrioventricular dehiscence. For the reconstruction we first reinforced the mitral annulus in segment A1-A3 using bovine pericardium patches (Fig. 1B). In the next step we used a doubled patch of bovine pericardium, anchored in the middle to the reconstructed mitral annulus and then with one half set in, to reconstruct and close the left atrial roof. The other half of the doubled patch was cut off. This double-patch technique allowed reconstruction of the aortic and the mitral annulus. In a full UFO procedure the ‘body’ (edge of the doubled patch) is fixed to the anterior mitral annulus. One wing of the butterfly is used to close the left atrial roof while the other wing can be used for reconstruction of the non-coronary sinus Valsalva. We implanted the ascending aorta graft, a custom-made biological composite valve graft (Fig. 1C). To construct this graft, we used a pericardium patch (10 cm x 16 cm) and sutured a bovine pericardial tube graft containing an aortic valve prosthesis (Trifecta™ 25 mm diameter) onto it. In our department the Trifecta™ valves are used as standard for patients aged > 70 years. The aortic root graft was proximally anastomosed by pericardium under layered Prolene sutures, which were stitched through the mitral valve neo-annulus (Fig. 2, Video 3). We then re-implanted the coronary buttons using the Bentall-de Bono technique [3]. The distal anastomosis of the 2/3 ascending aorta graft was also protected by pericardium patch-padded sutures. The left atrial roof was closed with a running suture (Fig. 1D). After thorough surgical haemostasis, the patient was weaned from cardiopulmonary bypass. The patient had a spontaneous sinus rhythm (Video 4). The clamping time was 149 min, the perfusion time 191 min. The thorax was closed using the standard technique.

### Follow-up

The patient was extubated on the first postoperative day. The fluorescence-in-situ-hybridization confirmed the presence of *Staphylococcus epidermidis*. An intraoperative smear of the abscess showed *Cutibacterium acnes* in the microbiological examination. The antibiotic therapy was supplemented with benzylpenicillin. A postoperative control echocardiogram confirmed a correct position and normal valve function without paravalvular leak of the newly implanted aortic valve prosthesis. The postoperative CT scan showed an adequate result.

The 3-year follow-up was uneventful. The latest echocardiography from October 2025 showed a normal left ventricular ejection fraction and a mean pressure gradient of 8 mmHg for the aortic valve prosthesis. The mitral valve showed a trivial central jet with a small morphological calcification of the posterior mitral leaflet. The IVFB appeared unremarkable. Quality of life was assessed using the Kansas City Cardiomyopathy Questionnaire (KCCQ-12). The patient’s results correspond to a good to excellent health status.

## Discussion

Prospectively, the number of patients with infective endocarditis will increase. Furthermore, infective endocarditis has become the leading indication for the removal of transcatheter aortic valves. Infection of the stent frame is more likely to lead to destructive abscesses. Therefore, the indication for complex surgical treatment will be more important in the near future [4].

The technique outlined in this work can be used for the surgical treatment of extensive aortic root endocarditis with a circular abscess located in the aortic valve anulus. The typical hemi-UFO procedure case presents with aortic root pathology extending into the IVFB, but sparing the mitral valve. Possible cases are endocarditis with abscess or aortic valve disease with calcified IVFB. In comparison to the UFO procedure, as a technique with double valve replacement, used in cases with an abscess located in the IVFB, the demonstrated modified hemi-UFO technique is a relatively simple procedure. It has been described for cases in which the IVFB requires reconstruction but the native mitral valve is preserved. The typical hemi-UFO patient shows an aortic root pathology extending into the IVFB, which is caused by endocarditis with an abscess or aortic valve/prosthetic disease with a calcified IVFB [5–7]. This technique can be thought of as an extended Manouguian procedure [1].

The risk of developing endocarditis may be higher for the spared mitral valve [5]. In terms of re-infection rates, reconstruction and replacement of infected tissue with biological pericardial conduits and suture pledgets seem to show a better outcome compared to artificial tissue [8, 9]. The best results can be assumed for using homografts, which however are not available in every centre. In case of abscess aortoventricular dehiscence, restoration of the structure and anchoring of the preserved mitral valve can be achieved by making use of the AML adhering to the aortic homograft [10].

The modified technique presented here not only allowed us to preserve the mitral valve and avoid the full UFO procedure, it also somewhat lowers some of the risk of the highly challenging UFO procedure for which previous studies have reported an operative mortality of up to 24% in patients with a paravalvular abscess [2, 11, 12]. The shorter operation time lowers the risk for operative and postoperative complications. The preservation of the mitral valve lowers the risk for postoperative iatrogenic type III atrioventricular block since mitral valve replacement is associated with higher risk of atrioventricular block.

The best argument in favour of the hemi-UFO procedure is the preservation of the mitral valve subvalvular apparatus and of the left ventricular geometry and function. In high-risk, multimorbid patients, this approach may prove beneficial when extensive and prolonged cardiac surgery is required.

While the use of custom-made bovine pericardial conduits can be superior with regard to the recurrence of infection, the long-term risks for aneurysmal dilatation and calcification can be higher in comparison with prosthetic conduits. There is a lack of data, and the available data which must be evaluated in the long term. Most of the data from large cohorts concerning handmade pericardial conduits were obtained in congenital heart surgery. A recent study by Yenduri et al. with 185 patients reported endocarditis in 4.3% of the patients (early in 3, late in 4). Twenty-two patients needed re-intervention with either dilatation (*n* = 18) or stenting (*n* = 4). Twenty-eight patients needed surgical replacement the conduit at a median of 37 (21–65) months [13].

There is a large retrospective study (*n* = 65), which compared prosthetic conduits with allograft conduits in patients with endocarditis and periannular abscess. The 11-year follow-up showed recurrent infection in 27.1% of the prosthetic group and 3.2% of the allograft group. The survival rate was 82.1% in the allograft group and 64.7% in the prosthetic group [14]. The patient’s EuroSCORE II of 50.64% indicates an extremely high operative risk. The indication for a UFO procedure is rare and is established in most cases as a last resort. There must be a prior heart team discussion in which the extent of the infection, patient comorbidities and possible outcome are discussed for best decision-making. Our report describes a single case, which limits the generalizability and means that conclusions cannot be drawn for every endocarditis patient due to the individual anatomical pathology.

## Supplementary Information

Below is the link to the electronic supplementary material.

Supplementary Material 1: Video 1: Preoperative transesophageal echocardiography. Short-axis (left), long-axis (right) view of the aortic prosthesis with large floating vegetations. Color Doppler shows severe aortic regurgitation and stenosis.

Supplementary Material 2: Video 2: Preoperative computed tomography angiography in transverse plane showing circular endocarditis of the aortic prosthesis with pleural effusions on both sides.

Supplementary Material 3: Video 3: Intraoperative view. Surgical step before the custom-made biological composite graft parachutes onto the aortic annulus. Visualization of the closed left atrial roof and direct view into the left ventricular outflow tract.

Supplementary Material 4: Video 4: Intraoperative view. Finished cardiopulmonary bypass. Visualization of the implanted custom-made biological composite graft.

## Data Availability

No datasets were generated or analysed during the current study.
